# A commentary on ‘Comparison of survival benefit and safety between surgery following conversion therapy versus surgery alone in patients with surgically resectable hepatocellular carcinoma at CNLC IIb/IIIa stage: a propensity score matching study’

**DOI:** 10.1097/JS9.0000000000001789

**Published:** 2024-06-10

**Authors:** Jia Luo, Qian Yang, Wen Ma, Hua Fan

**Affiliations:** Department of Oncology and Hematology, The People’s Hospital of Leshan, Leshan, Sichuan Province, People’s Republic of China

Most patients with hepatocellular carcinoma (HCC) are often diagnosed at an advanced stage owing to its insidious onset, consequently forfeiting the chance for radical resection. In recent years, conversion therapy has gained extensive attention as a promising treatment strategy for these patients with initially unresectable HCC (uHCC), offering an improved prognosis^1,2^. Conceptually, conversion therapy involves the conversion of an uHCC into resectable HCC followed by surgical removal, with the ultimate goal of prolonging survival^3^. However, the optimal candidate population, precise regimen, and efficiency of conversion therapy remain ambiguous owing to significant heterogeneity across various studies. A single-arm, multicenter, prospective study evaluated the efficacy of transcatheter arterial chemoembolization (TACE) combined with lenvatinib and camrelizumab as conversion therapy for uHCC, demonstrating that this triple therapy had a high conversion efficiency (30 out of 55 patients, 54.5%) and manageable safety^4^. In another single-arm, phase 2 trial, the sequential use of TACE and stereotactic body radiotherapy followed by immunotherapy as conversion therapy for locally advanced uHCC, demonstrated a conversion efficiency of 55% (18 out of 33 patients)^5^.

We read with great interest a retrospective propensity-matched analysis by Ma *et al*.^6^ published in the International Journal of Surgery, which explored the survival benefit and safety of surgery following conversion therapy in patients with surgically resectable HCC at the China Liver Cancer Staging (CNLC) IIb/IIIa stage. The study included 95 patients and concluded that the group undergoing conversion therapy demonstrated a significantly longer median recurrence-free survival (17.1 vs. 7.0 months; *P*=0.014) and a lower incidence of microvascular invasion (23.3 vs. 81.5%; *P*<0.001) compared to the group who underwent surgery alone. Although the findings of the study are crucial in elucidating the prominent role of conversion therapy in the comprehensive treatment of HCC, there are still several issues worthy of further exploration.

First, the authors elaborated on the concept of conversion therapy and defined surgical resectability to distinguish patients with surgically resectable but oncologically uHCC. However, these surgically resectable cases without portal vein tumor thrombus (PVTT) or with PVTT grade 1 to 2 are more likely to be considered as initially resectable, and the preoperative adjuvant therapy is more appropriately termed neoadjuvant therapy^3^. Distinct from conversion therapy, the primary aim of neoadjuvant therapy is to reduce the risk of postoperative recurrence in patients with initially resectable HCC who have high-risk factors for recurrence. Notably, in patients with surgically resectable but locally advanced HCC, conversion therapy and neoadjuvant therapy partially overlap in their meanings and have the same ultimate goal. Second, the authors included only patients who completed R0 resection and excluded those who lost the opportunity for surgery owing to disease progression during conversion therapy or who did not achieve R0 resection. This approach potentially mitigates the risks associated with conversion therapy and may overestimate its effectiveness. Third, a significant proportion of patients in both groups received postoperative adjuvant therapy. Although details of the adjuvant therapy were not provided, it is probable that the conversion therapy group adhered to the previously effective conversion therapy regimen, while the surgery alone group used an unproven regimen. Consequently, despite the absence of discernible variations in clinicopathological characteristics between the two groups, this factor alone inevitably increased the survival period of the experimental group, leading to biased results. Fourth, the starting point for defining recurrence-free and overall survival was the date of surgery, not the date of the initial treatment. Since the combined duration of conversion therapy and preoperative waiting is typically 2–4 months, this definition actually underestimates the true survival of the conversion therapy group. Fifth, as mentioned by the authors, the diversity of conversion therapy strategies limits the further generalization of the findings of this study. Although the conversion therapy protocols and cycles for each patient were presented, the inclusion of intervals between therapies could enhance the informativeness of the data, given the incomplete equivalence of treatment cycles and intervals. Finally, the evaluation of oncological resectability lacks clear and unified standards, and the multidisciplinary team (MDT) approach is undoubtedly the most effective method at this stage. In this study, a MDT was utilized for the assessment of resectability, formulation of therapy protocols, and determination of surgical timing, which carries significant implications for guiding future research in this area.

Overall, we commend the authors for their remarkable efforts in assessing the survival benefits and safety of preoperative conversion therapy in patients with surgically resectable HCC. Their work significantly contributes to our understanding of this complex treatment approach. However, more prospective clinical trials are needed to determine the optimal regimen and indications for conversion therapy, making it a promising treatment strategy for the multidisciplinary management of advanced uHCC.

## Ethical approval

Not applicable for this study.

## Source of funding

The comment dose not receive any funding.

## Author contribution

J.L.: study design and writing; Q.Y. and W.M.: critical review; H.F.: study supervision.

## Conflicts of interest disclosure

The authors declare that they have no conflict of interest.

## Research registration unique identifying number (UIN)

None.

## Guarantor

Jia Luo.
